# Limitations of the transmitted photonic spin Hall effect through layered structure

**DOI:** 10.1038/s41598-021-00681-0

**Published:** 2021-10-26

**Authors:** Chong Miao, Dongxue Wang, Eric Herrmann, Zhiyuan Zheng, Haochong Huang, Hua Gao

**Affiliations:** 1 School of Science, China University of Geosciences, Beijing, 100083 China; 2 Department of Materials Science and Engineering, College of Engineering, University of Delaware, Newark, DE 19716 USA

**Keywords:** Optics and photonics, Optical materials and structures, Optical physics

## Abstract

In this paper, we show theoretically that the spin-dependent transverse shift of the transmitted photonic spin Hall effect (SHE) through layered structure cannot exceed half of the incident beam waist. Exact conditions for obtaining the upper limit of the transmitted SHE are clarified in detail. In addition, different from the popular view in many investigations, we find that there is no positive correlation between the spin-dependent transverse displacement and the ratio between the Fresnel transmission coefficients (t_p_, t_s_). In contrast, the optimal transmission ratio is determined by the incident angle and the beam waist. Moreover, two conventional transmission structures are selected and studied in detail. The characteristics of the transverse displacements obtained are in very good agreement with our theoretical conclusions. These findings provide a deeper insight into the photonic spin Hall phenomena and offer a guide for future related research.

## Introduction

When linearly polarized light is incident upon a structure, the opposite spin components (left and right circular polarizations) of the reflected/transmitted beam will shift toward opposite directions perpendicular to the incident plane, this phenomenon is called the photonic spin Hall effect (SHE)^1–3^. The photonic SHE not only exhibits potential applications in precision metrology and also offers new opportunities for controlling photons in nanophotonic devices. Therefore, the photonic SHE has received considerable attention over the past two decades. However, the photonic SHE is typically an insignificant phenomenon; the spin-dependent spatial splitting is usually limited by a fraction of the incident wavelength^3–6^. Thus, to promote its application, exploring enhanced photonic SHE has become especially necessary. In recent years, various resonant mechanisms have been proposed to enhance the photonic SHE which include surface plasmon resonance (SPR)^7^, long range SPR excitation^8^, photon tunneling^9^, resonant optical tunneling effect (ROTE)^10^, frustrating total internal reflection (FTIR)^11^, bound states in the continuum (BICs) ^12^, graphene/MoS_2_ heterostructures^13^, waveguide^14^, etc. On the other hand, a variety of unconventional materials have also been proposed to enhance the photonic SHE, including epsilon-near-zero (ENZ) materials^15^, anisotropic ENZ^16^, hyperbolic metamaterials^17,18^, anisotropic metamaterials^19^, graphene^20–22^ and black phosphorus^23^, polymers^24^, topological insulators^25^, and even transversally disordered media^26^ which present great difference from the other photonic SHE materials, for example, they naturally display spin-dependent shifts of larger magnitude which is proportional to the transverse wavelength rather than the wavelength itself. However, most of the examples mentioned above are only theoretical studies. In experiment, the earlier significantly enhanced photonic SHE appeared in the reflection near the Brewster angle^27,28^. The observed transverse displacement is up to several microns, which is dozens of times larger than its previously reported observations. Several years ago, Takayama et al. reported a new experimental record of the transverse spin shift. In their report, the transverse spin shifts as large as a few hundred microns were observed through a multilayer hyperbolic metamaterial^18^. Recently, Dai et al. reported an even larger photonic SHE that can be directly distinguished by human eyes^29^. By exciting resonant ultrahigh-order modes inside a birefringent symmetrical metal cladding planar waveguide, they measured the transverse spin shift from the waveguide surface at near-normal incident angles and realized a transverse shift at the order of sub-millimeter. This work has taken another important step in the experimental exploration of the photonic SHE.

Although many instances of enhanced SHE have been obtained, at present, many researchers continue striving for further enhancement of the spin-dependent spatial splitting. This raises the question: To what extent can the photonic SHE be enhanced? How much room is left for further improvement? Besides, it is known that the Fresnel transmission coefficients play a vital role on the photonic SHE. In many works, the authors made efforts to improve the Fresnel transmission ratio to increase the spatial splitting^10,15,22,30^. Another question then arises: Does an increase in the ratio of the Fresnel transmission coefficients really lead to an increase of the transverse shift? In addition, because the photonic SHE is a result of the interaction between the incident light and the structures, the incident beam also plays an important role on the value of the transverse shift. It has been shown that the transverse displacements are different for different beam waists^14^. Thus, what role does the beam waist play in the transverse displacement?

In this paper, the above questions are studied by theoretically deducing and analyzing the spin-dependent transverse displacement of a transmitted photonic SHE. It is found that for all layered structures, including all kinds of resonant structures and various materials, their spin splitting has an upper limit, i.e., the transverse shift cannot be enhanced arbitrarily. In addition, the transverse shift of the photonic SHE is related to the beam waist of incident light. We find that the transverse shift increases with increasing beam waist. Moreover, the transverse shift does not always increase with the increase of the transmission ratio. For a given beam waist, the optimal transmission ratio is dependent on the incident angle. We verify the above conclusions by studying the transmitted photonic SHEs in both an ENZ slab and an optical resonant tunneling structure. These findings provide deeper insight into the optical spin-dependent splitting phenomena and can better guide the study of the photonic SHE.

## Upper limit of the transmitted photonic SHE through layered structure

In the literature, there are several different analytical expressions for the transverse displacement of the transmitted photonic SHE, for example, Eqs. (18) and (19) in Ref.^30^, Eq. (7)  in Ref.^22^, Eq.  (5) in Ref.^15^ and so on. Through careful comparison, we found that these formulas cannot be transformed and unified with each other. Therefore, it is necessary to derive the formulas of the transmitted transverse displacements again. We use the same theoretical method which has been used in most papers. The transverse displacements for H and V polarized light are obtained respectively as (See supplementary information for detailed derivation process):

1 $${\delta }_{H}^{\pm }=\pm \frac{{k}_{0}{\omega }_{0}^{2}cot{\theta }_{i}[{\left|{t}_{p}\right|}^{2}-Re\left({t}_{p}\cdot {{t}_{s}}^{*}\right)]}{{{k}_{0}}^{2}{\omega }_{0}^{2}{\left|{t}_{p}\right|}^{2}+{{\left|{t}_{p}-{t}_{s}\right|}^{2}cot}^{2}{\theta }_{i}+\frac{{\partial t}_{p}}{\partial {\theta }_{i}}\cdot \frac{{{\partial t}_{p}}^{*}}{\partial {\theta }_{i}}}$$

2 $${\delta }_{V}^{\pm }=\pm \frac{{k}_{0}{\omega }_{0}^{2}\mathit{cot}{\theta }_{i}\left[{\left|{t}_{s}\right|}^{2}-\mathit{Re}\left({t}_{s}\cdot {{t}_{p}}^{*}\right)\right]}{{k}_{0}^{2}{\omega }_{0}^{2}{\left|{t}_{s}\right|}^{2}+{{\left|{t}_{s}-{t}_{p}\right|}^{2}\mathit{cot}}^{2}{\theta }_{i}+\frac{\partial {t}_{s}}{\partial {\theta }_{i}}\cdot \frac{\partial {t}_{s}^{*}}{\partial {\theta }_{i}}}$$

One can see from Eqs. (1) and (2) that the transverse displacements depend on the beam waist ω_0_, incident angle θ_i_, and Fresnel transmission coefficients t_p_ and t_s_. Selecting Eq. (2) as an example, we investigate these dependent properties in detail. We suppose that:

3 $$\frac{{t}_{p}}{{t}_{s}}=r{e}^{i\varphi },\mathrm{ r}\ge 0,\mathrm{ \varphi }\in [-\pi ,\pi ]$$

where r and φ are the absolute value of the ratio and phase difference between the Fresnel transmission coefficients, respectively. Then,

4 $${t}_{p}=r{t}_{s}{e}^{i\varphi }=r{t}_{s}\left(\mathit{cos}\varphi +i\mathit{sin}\varphi \right)$$

By substituting Eq. (4) into Eq. (2), the transverse shifts of V polarization can be calculated as

5 $${\delta }_{V}^{\pm }=\pm \frac{{k}_{0}{\omega }_{0}^{2}{\left|{t}_{s}\right|}^{2}\left(1-r\,\mathit{cos}\varphi \right)\mathit{cot}{\theta }_{i}}{{k}_{0}^{2}{\omega }_{0}^{2}{\left|{t}_{s}\right|}^{2}+{\left|{t}_{s}\right|}^{2}\left(1-2r\,\mathit{cos}\varphi +{r}^{2}\right){\mathit{cot}}^{2}{\theta }_{i}+\frac{\partial {t}_{s}}{\partial {\theta }_{i}}\cdot \frac{\partial {t}_{s}^{*}}{\partial {\theta }_{i}}}$$

The absolute value of the transverse shift is then

6 $$\left|{\delta }_{v}\right|=\frac{{{k}_{0}\omega }_{0}^{2}\left|\left(1-r\,\mathit{cos}\varphi \right)\right|\mathit{cot}{\theta }_{i}}{{k}_{0}^{2}{\omega }_{0}^{2}+{\left(1-r\,\mathit{cos}\varphi \right)}^{2}{\mathit{cot}}^{2}{\theta }_{i}+{r}^{2}{sin}^{2}\varphi {\mathit{cot}}^{2}{\theta }_{i}+\frac{\partial {t}_{s}}{\partial {\theta }_{i}}\cdot \frac{\partial {t}_{s}^{*}}{\partial {\theta }_{i}}/{\left|{t}_{s}\right|}^{2}}$$

We now analyze which conditions must be satisfied to obtain as large a transverse shift as possible. First, $\frac{\partial {t}_{s}}{\partial {\theta }_{i}}=0$ is available when t_s_ is a constant or has an extreme value (resonance occurs). Thus, $\frac{\partial {t}_{s}}{\partial {\theta }_{i}}=0$ is a condition to enhance the transverse shift. Second, if $\mathit{sin}\varphi =0$ can be satisfied, the value of the transverse displacement will become even larger. However, this assumption does not affect the value range of $r\,\mathit{cos}\varphi$ because it contains a coefficient r. When $\mathit{sin}\varphi =0$, it can be considered that the term $r\,\mathit{cos}\varphi$ is independent of $\mathit{sin}\varphi$. Thus, the transverse shift can be further enhanced to

7 $$\left|{\delta }_{v}\right|=\frac{{{k}_{0}\upomega }_{0}^{2}\left|\left(1-r\,\mathit{cos}\varphi \right)\right|\mathit{cot}{\theta }_{i}}{{\mathrm{k}}_{0}^{2}{\upomega }_{0}^{2}+{\left(1-\mathrm{rcos\varphi }\right)}^{2}{\mathrm{cot}}^{2}{\uptheta }_{\mathrm{i}}}$$

Through simple mathematical analysis, we find that $\left|{\delta }_{v}\right|$ has a maximum value of ω_0_/2 if the condition $1-rcos\varphi =\pm {k}_{0}{\omega }_{0}tan{\theta }_{i}$ can be satisfied in Eq. (7). Considering r > 0, if $\mathrm{cos}\varphi =1$, the optimal transmission ratio is $r={k}_{0}{\omega }_{0}tan{\theta }_{i}-1$. If $\mathrm{cos\varphi }=-1$, the optimal transmission ratio may take one or two values of $r=\pm {k}_{0}{\omega }_{0}tan{\theta }_{i}+1$.

It is clear that the upper limit for the transverse shift is half of the beam waist. Therefore, after transmission through any layered structure or any material slab, the spin-dependent spatial splitting of a linearly polarized Gaussian beam cannot exceed its beam waist ω_0_. It is worth noting that the upper limit can only be reached when the Fresnel transmission coefficients satisfy certain conditions, which include:(a) $\frac{\partial {\mathrm{t}}_{\mathrm{s}}}{\partial {\uptheta }_{\mathrm{i}}}\cdot \frac{\partial {\mathrm{t}}_{\mathrm{s}}^{*}}{\partial {\uptheta }_{\mathrm{i}}}=0$; (b) $\mathrm{sin\varphi }=0$; (c) The optimal transmission ratio is obtained, i.e., when $\mathrm{cos\varphi }=1$,$\mathrm{r}=\pm {\mathrm{k}}_{1}{\upomega }_{0}\mathrm{tan}{\uptheta }_{\mathrm{i}}+1$; when $\mathrm{cos\varphi }=-1$, $\mathrm{r}={\pm \mathrm{k}}_{1}{\upomega }_{0}\mathrm{tan}{\uptheta }_{\mathrm{i}}-1$. In fact, using Eq. (3), condition (b) and condition (c) can be further unified into one condition of $\frac{{\mathrm{t}}_{\mathrm{p}}}{{\mathrm{t}}_{\mathrm{s}}}={\pm \mathrm{k}}_{0}{\upomega }_{0}\mathrm{tan}{\uptheta }_{\mathrm{i}}-1$. Thus, for a certain incident wavelength, the optimal transmission ratio of $\frac{{\mathrm{t}}_{\mathrm{p}}}{{\mathrm{t}}_{\mathrm{s}}}$ is determined by the incident angle and the beam waist.

**Figure 1:**
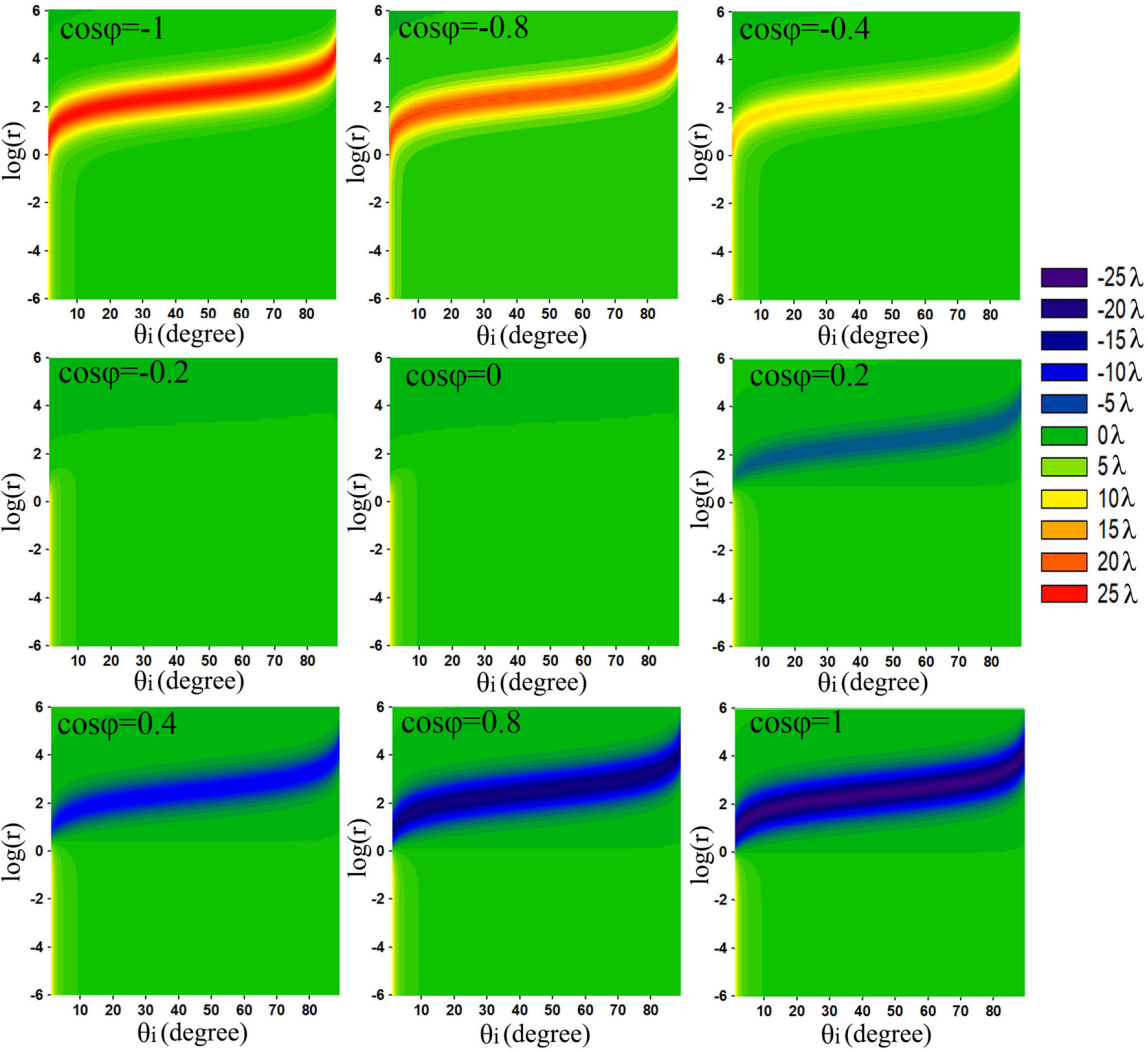
Dependence of the transverse shifts on θ_i_ and log(r) at different values of cosφ, where ω_0_ = 50λ.

Now, using Eq. (5), we numerically analyze dependencies of the transverse displacement on the absolute value of the transmission ratio r, phase difference φ, and incident angle θ_i_. Here, we do not consider any specific transmission structure or material, so it is supposed that r can take any positive value for any incident angle. In the denominator, the term $\frac{\partial {\mathrm{t}}_{\mathrm{s}}}{\partial {\uptheta }_{\mathrm{i}}}\cdot \frac{\partial {t}_{s}^{*}}{\partial {\theta }_{i}}$ is usually much smaller than the first two terms, thus, in calculation this term is neglected. Figure 1 gives the calculated ${\updelta }_{\mathrm{v}}^{+}$ as functions of log(r) and the incident angle θ_i_ for different $\mathrm{cos\varphi }$ for a certain beam waist of 50λ. Clearly, the distributions of ${\updelta }_{\mathrm{v}}^{+}$ change as $\mathrm{cos\varphi }$ varies in different subgraphs. The closer the absolute value of $\mathrm{cos\varphi }$ is to 1, the greater the maximum absolute value of ${\updelta }_{\mathrm{v}}^{+}$ is. When the absolute value of $\mathrm{cos\varphi }$ is equal to 1, the maximum shift almost reaches its upper limit ω_0_/2. On the contrary, if $\left|\mathrm{cos\varphi }\right|<1$, no matter what value r takes, the transverse shift cannot reach its upper limit. Therefore, the phase difference plays a crucial role on the transverse shift. In addition, for a certain $\mathrm{cos\varphi }$, the maximum transverse shift is obtained at different log(r) for different incident angles, where log(r) slightly increases with the incident angle. For a certain incident angle, the optical value of r (corresponding to the largest transverse shift) is almost the same for different $\mathrm{cos\varphi }$. Thus, the optimal value of r is mainly determined by the incident angle while is irrelevant to $\mathrm{cos\varphi }$.

**Figure 2:**
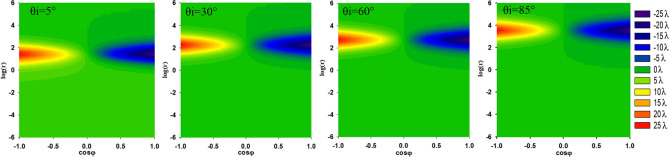
Dependence of transverse shifts on $\mathrm{cos\varphi }$ and log(r) at different incident angles, where ω_0_ = 50λ.

Figure 2 is the transverse shift as functions of $\mathrm{cos\varphi }$ and log(r) for several different incident angles. It exhibits similar features to those of Fig. 1: The upper limit ω_0_/2 of the transverse shift can be obtained at $\mathrm{cos\varphi }$= ± 1 when log(r) takes certain values. With $\mathrm{cos\varphi }$ deviating from ± 1, the largest attainable transverse shift becomes smaller and smaller. For different incident angles, the largest transverse shifts appear at different log(r). When the incident angle increases, log(r) of the largest transverse shift increases, too. For a certain incident angle, the blue ribbon is slightly higher than the red ribbon, meaning that the negative transverse shift needs slightly larger r than that of the positive transverse shift. For example, the largest negative transverse shift and the largest positive transverse shift, which correspond to $\mathrm{cos\varphi }=1$ and $\mathrm{cos\varphi }=-1$, appear at $r={k}_{0}{\omega }_{0}tan{\theta }_{i}+1$ and $r={k}_{0}{\omega }_{0}tan{\theta }_{i}-1$, respectively.

**Figure 3:**
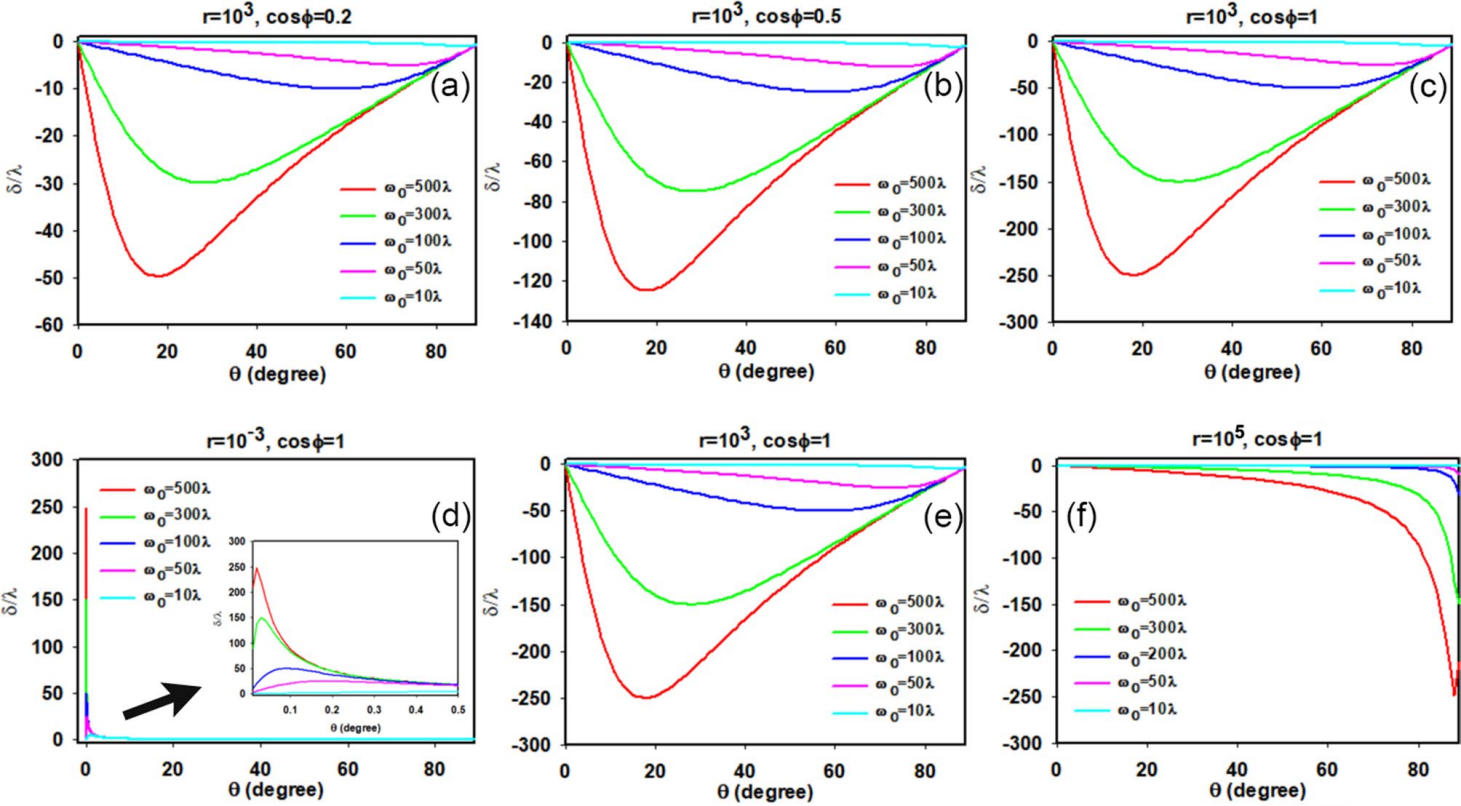
Dependence of the transverse shifts on ω_0_ at certain r and different $\mathrm{cos\varphi }$ **(a–c)**; dependence of the transverse shifts on ω_0_ at certain $\mathrm{cos\varphi }$ and different r **(d–f)**.

The incident beam is also an important factor that can influence the transverse shift. Figure 3 shows the transverse shifts varying with the incident angle for different beam waists, where the first row corresponds to the same r and different $\mathrm{cos\varphi }$, while the second row corresponds to different r and the same $\mathrm{cos\varphi }$. Apparently, for all the figures, the larger the beam waist is, the larger the maximum transverse shift is. This property can also be easily deduced from the transverse shift formula, Eq. (6). In Fig.3a–c, all the curves of the transverse shifts varying with the incident angle are similar. The larger the absolute value of $\mathrm{cos\varphi }$ is, the greater the transverse shift is. However, for a certain value of $\mathrm{cos\varphi }$, as shown in Fig.3d–f, the largest shifts for different r are obtained at different incident angle. When r takes a small value of 10^–3^, the largest shift appears at a very small incident angle, as shown in Fig. 3d, while for a large value of 10^5^, the largest shift moves to a very large incident angle, almost to 90 degrees. For a special value of $\mathrm{cos\varphi }=1$, the largest shift values for different r all reach their upper limits of ω_0_/2. Therefore, there is no positive correlation between the transverse shift and the ratio r.

The above analyses are not limited to a specified structure as only the Fresnel coefficients, t_s_ and t_p_, are used in the derivation. Actually, besides layered optical structures, many periodic and patterned structures as long as their transmission Fresnel coefficient can be characterized by uniform t_s_ and t_p_, the upper limit is appliable. However, for uneven interfaces, for example, gradient index material or gradient geometric phase metasurface, the upper limit should be reconsidered. Besides, as the upper limit is not based on a specified structure, the parameters r and φ can be supposed to take any reasonable value. However, for a specific transmission structure, when the incident angle θ_i_ is given, the Fresnel transmission coefficients are both known. If these known values satisfy the above conditions, the transverse shift can reach its upper limit. In contrast, if the incident angle θ_i_, the value of the transmission ratio r, and the phase difference φ cannot satisfy the needed conditions, the upper limit is not attainable.

## The properties of transmitted photonic SHE can be confirmed by physical structures

**Figure 4:**
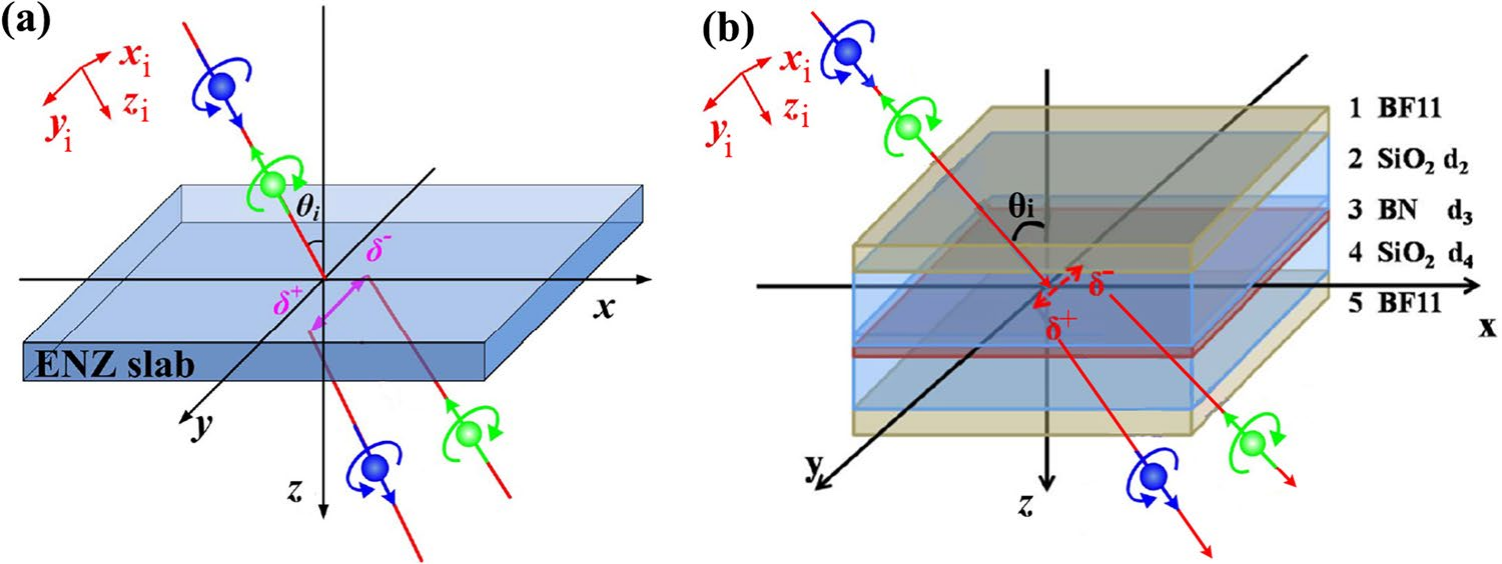
Illustration of the transmitted photonic SHE through an ENZ slab **(a)** and a ROTE structure **(b)**.

To validate the above conclusions, here we select two typical photonic SHE enhanced structures and investigate their transverse shifts. One method considers an ENZ slab, and the other is a ROTE structure. Figure 4a,b give the illustrations of the photonic SHE through an ENZ and a ROTE structure, respectively. They schematically show that when a linearly polarized beam is launched into an ENZ slab/a ROTE structure, the transmitted light beam splits into right and left circular polarized components, which undergo positive and negative transverse displacements, respectively. Now we calculate their transverse shifts and analyze their upper limits and the corresponding parameter dependent properties. In calculation the Fresnel transmission coefficients are calculated by using the transfer matrix method^31^, and then the amplitudes and the arguments of the coefficients can both be extracted to determine r and φ.

**Figure 5:**
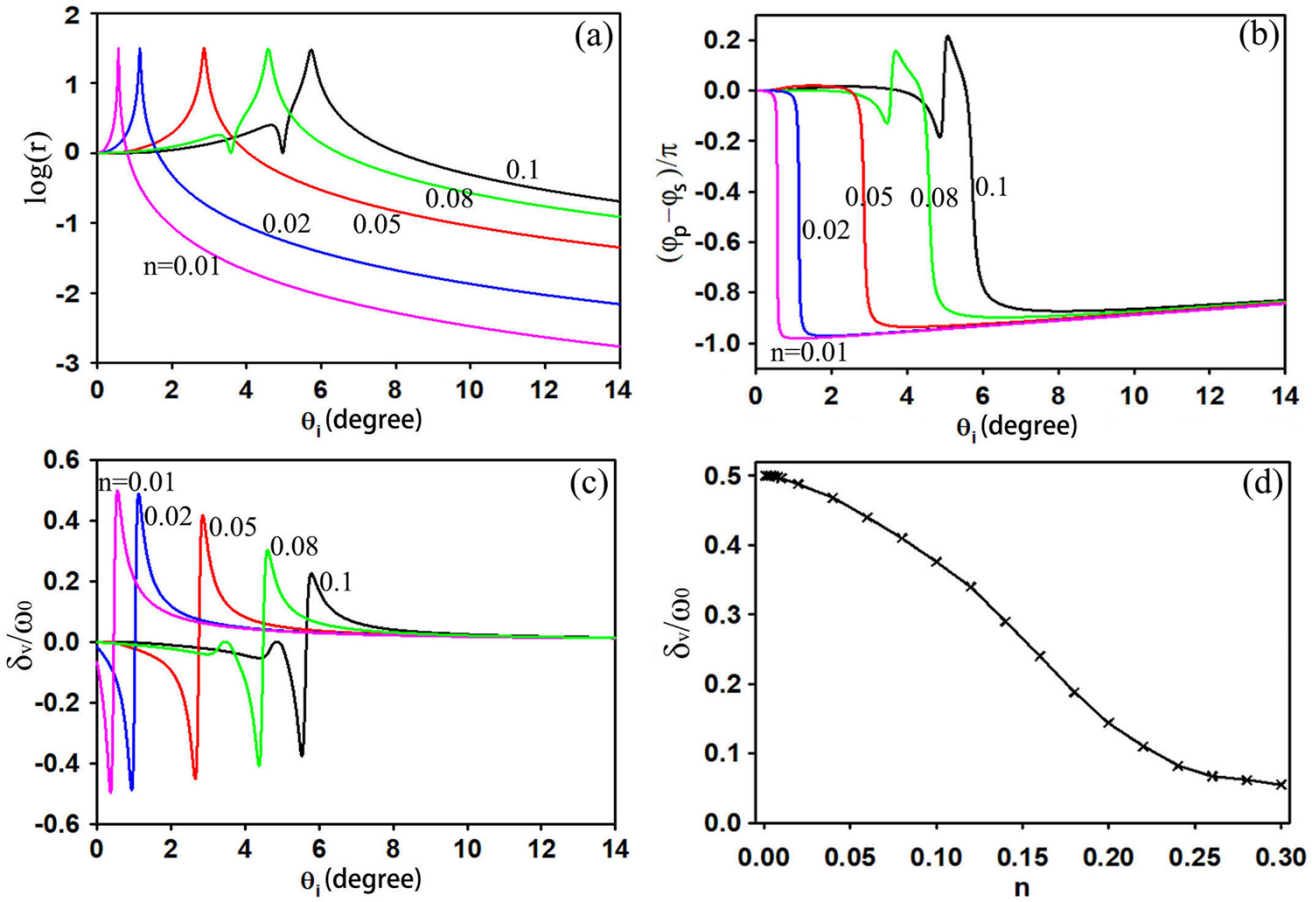
Log(r) **(a)**, phase difference **(b)**, and transverse shifts **(c)** of V-polarized input as functions of incident angle for different refractive indices of the ENZ slab. **(d)** Maximum transverse shift varying with the refractive index of the ENZ slab. The parameters of the ENZ slab and incident beam are chosen as h = 3λ, $\mathrm{n}=\sqrt{\upvarepsilon }$, µ = 1, and ω_0_ = 50λ.

In the ENZ slab calculations, its permeability is set to 1 and the thickness is set to h = 3λ. In order to be consistent with the theoretical analysis, a linear V-polarized Gaussian beam with abeam waist of 50λ is used as the incident light. Figure 5 gives the calculated critical parameters as functions of the incident angle for different refractive index n ($\mathrm{n}=\sqrt{\upvarepsilon }$). According to Ref.^15^, the Fresnel transmission coefficient t_p_ undergoes a distinct resonance near the Brewster angle, where its absolute value is greatly enhanced and is accompanied by an abrupt phase change of π. Due to the resonance of t_p_, the absolute value of transmission ratio r also undergoes a resonance and the phase difference experiences a change of π near the Brewster angle. In Fig. 5, it can be seen that the smaller the refractive index is, the smaller the resonance angle is, and the larger the attainable transverse shift. Figure 5d shows how the maximum transverse shift varies with the refractive index. In order to make the results more reliable, we decrease the interval of the refractive index when n tends to zero. As shown in Fig. 5d, the maximum transverse shift asymptotically approaches half of the beam waist as the refractive index approaches zero. This property is in very good agreement with the theoretical analysis. The properties of transmitted photonic SHE can be confirmed by physical structures.

**Figure 6:**
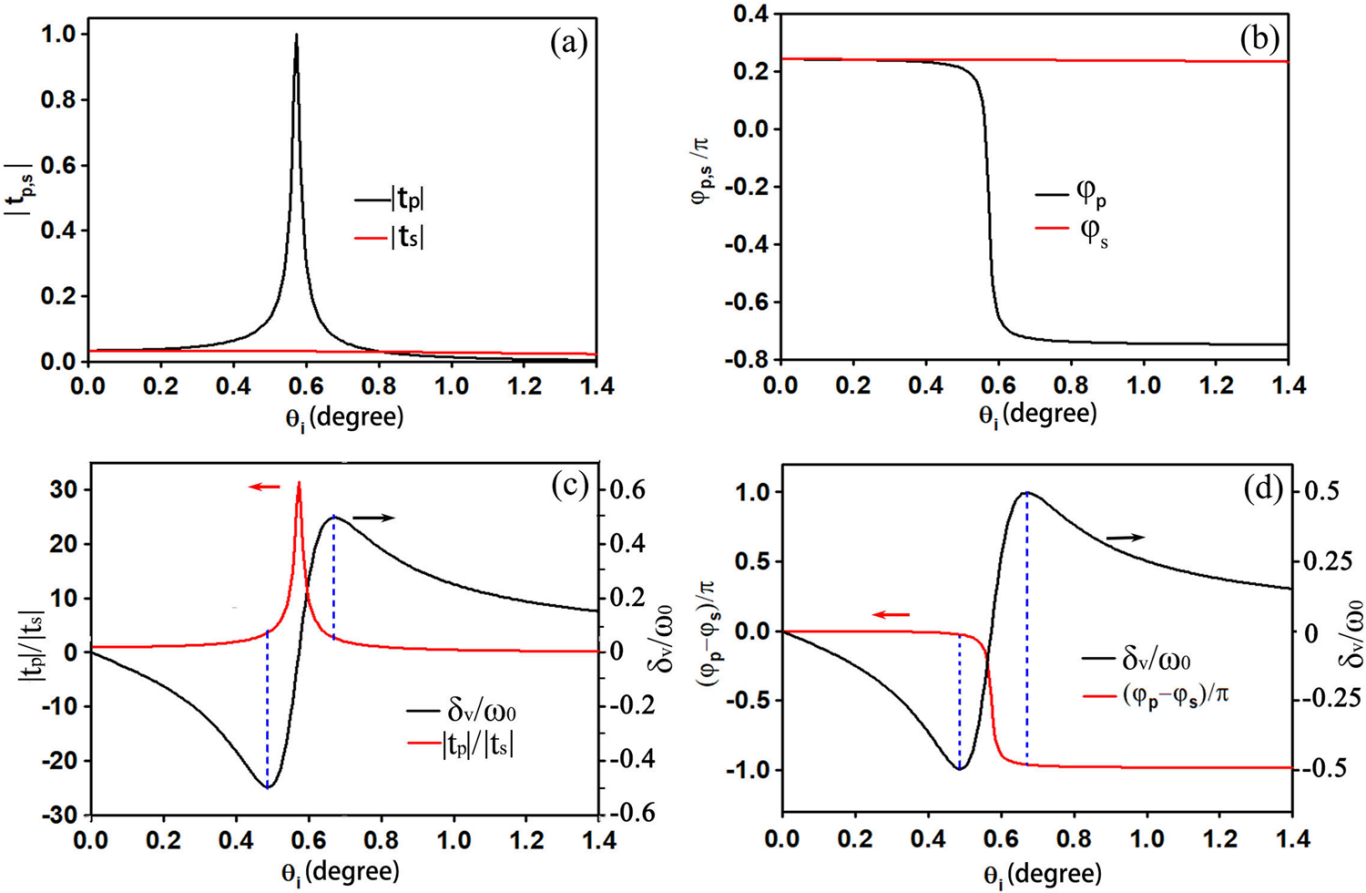
Amplitudes **(a)** and phase distributions **(b)** of the Fresnel transmission coefficients for the ENZ slab, **(c)** is the transverse shift and ratio of the Fresnel transmission coefficients near the Brewster angle, **(d)** is the transverse shift and phase difference distributions near the Brewster angle, where the thickness of the ENZ slab is 3λ, the refractive index is n = 0.01and the incident light is V-polarized Gaussian beam with a beam waist of 50λ.

To make clear the relationship between the transmission ratio r and the transverse shift, we magnify the resonance span for each refractive index. Figure 6 shows the details related to the Fresnel transmission coefficients and the transverse shift near the Brewster angle for n = 0.01. Figure 6a,b display the amplitude and phase properties of the transmission coefficients, respectively. It is clear that t_p_ undergoes a Lorentz resonance while t_s_ remains nearly unchanged. Thus, the condition of $\frac{\partial {\mathrm{t}}_{\mathrm{s}}}{\partial\uptheta }\cdot \frac{\partial {t}_{s}^{*}}{\partial \theta }=0$ is met within this range of incident angles. Figure 6c shows the distributions of the transmission ratio (red curve) and the transverse shift (black curve) near the resonance. Unlike the conclusions drawn in other investigations, the largest transverse shifts obtained here appear at a very small transmission ratio. As indicated by two blue dashed lines, the ratios of the Fresnel transmission coefficients are near zero. According to the theoretical analysis, these two small ratio values are reasonable because their incident angles are both very small. Figure 6d gives the distributions of the phase difference (red curve) and the transverse shift (black curve). The positive and negative largest values of the transverse shifts, indicated by the blue dashed lines, align almost with phase differences of -π and 0, respectively. Phase differences of -π and 0 satisfy cosφ = -1 or cosφ = 1, which is also in good agreement with the theoretical analysis. In addition, through comparison, we found that the transmission ratios corresponding to the maximum shifts vary with refractive index. Because the resonant incident angle increases with refractive index, the transmission ratio also increases with refractive index. Furthermore, the phase differences corresponding to the maximum transverse shifts are slightly closer to 0 and -π when the refractive index decreases, as can be seen from Fig. 5b. This is the critical reason why the transverse shifts increase when the refractive index decreases. However, even when decreasing the refractive index, the upper limit of the transverse shift cannot be exceeded.

**Figure 7:**
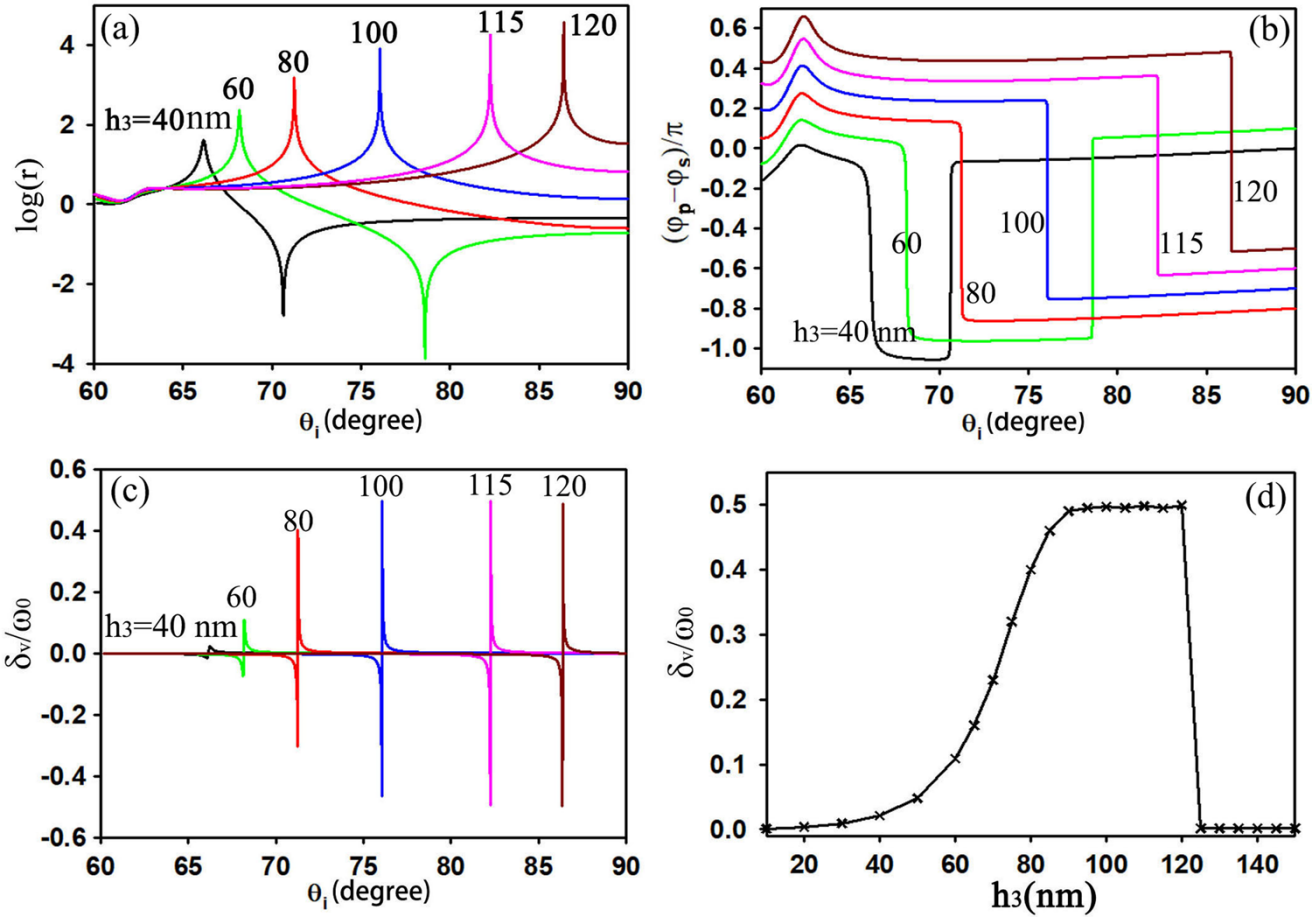
Log(r) **(a)**, phase difference **(b)**, and transverse shifts **(c)** of V-polarized input as functions of incident angle for different thicknesses of the BN. **(d)** The maximum transverse shift varying with the thickness of the BN. The incident light is V-polarized beam with a beam waist of 50λ, the structural and material parameters of the ROTE structure are the same in Ref.^10^ except h_3_ is changing.

As for the ROTE structure, we use the same structure proposed in Ref.^10^, where we consider structural and material parameters identical to those used in Fig. 6 of Ref.^10^, except the thickness of the boron nitride (BN). Figure 7a–c show the transmission ratios, phase differences and transverse shifts varying with the incident angle for different thicknesses of the BN. For clarity, the phase difference distributions in Fig. 7b are shifted upward by 0.1 for each thickness. Because the phases of t_p_ and t_s_ both experience an abrupt change of π when they are on resonance, their phase difference changes at every resonant peak/valley of r, resulting in a phase difference of π, too. Similar to the results in Ref.^10^, as the BN thickness increases, the transmission ratio, resonant incident angle and transverse shift increase gradually. Apparently, the transverse shift cannot increase sustainably. When the thickness of the BN approaches 100 nm, the transverse shift gradually tends to a constant value, which is just the upper limit of the transverse shift. Figure 7d gives the maximum transverse shift as a function of the BN thickness. When the thickness of the BN is small, the resonant tunneling effect onsets gradually and the maximum transverse shift increases gradually, too. While the optical tunneling effect resonates stronger, the maximum shift becomes larger and eventually approaches its upper limit. If the thickness is further increased, the resonant condition can no longer be satisfied and the resonant tunneling effect disappears, leading to a dramatic reduction of the maximum transverse shift. The curve showing the variation of the transverse shift with BN thickness, Fig. 7d, provides further evidence that there exists an upper limit to the transmitted photonic SHE and that its value is half of the beam waist.

**Figure 8:**
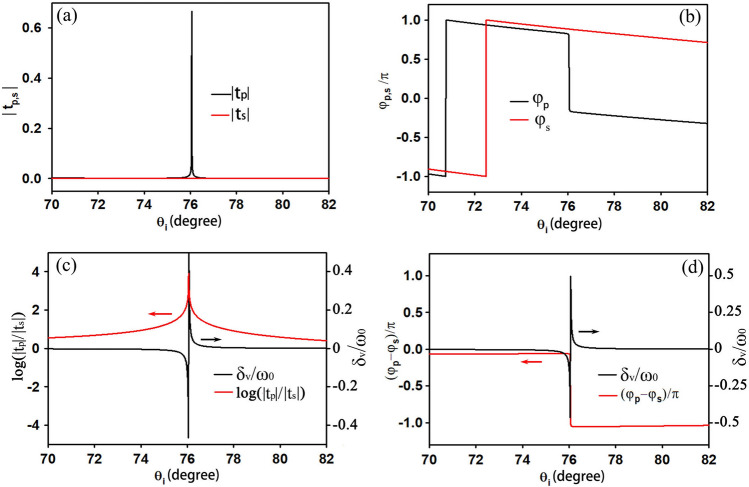
Amplitudes **(a)** and phase distributions **(b)** of the Fresnel transmission coefficients for a ROTE structure, **(c)** is the transverse shift and transmission ratio near the Brewster angle, **(d)** is the transverse shift and phase difference distributions near the Brewster angle, where the incident light is a V-polarized beam with a beam waist of 50λ.The structural and material parameters of the ROTE structure are the same in Ref.^10^ except h_3_ = 100 nm.

Similar to Fig. 6, in Fig. 8 we present magnified plots near the resonant incident angle for a specific BN thickness of 100 nm, where there is an apparent resonance of t_p_ while the t_s_ distribution remains relatively flat. Like the ENZ slab we analyzed earlier, when a resonance of t_p_ occurs, its phase distribution exhibits an abrupt change of π, around 76 degrees. Compared to the ENZ slab, the resonant quality of this ROTE structure is much higher, and its resonant width is extremely narrow. From Fig. 8c, it can be seen that the largest transverse shifts occur near the maximum of the transmission ratio r, which approaches 10^4^. In both the ENZ slab and ROTE structures, the upper limit of the transverse shifts is achieved. However, the incident angle of the largest transverse shifts in the ENZ slab is around 0.6 degrees, requiring the transmission ratio to be near-zero, while the resonant angle of the ROTE structure is around 76 degrees, requiring a very large transmission ratio, near 10^4^. These two cases both agree well with the theoretical analysis. Therefore, the expectation that "A large ratio would extremely enhance the transverse displacement of reflected (transmitted) light" in Refs. ^30^ and ^22^ may not be realized for small incident angles. As analyzed before, for a fixed wavelength and beam waist, the optimal transmission coefficient ratio is determined by the incident angle: the larger the incident angle, the larger the optimal transmission coefficient ratio becomes.

## Discussion

The value of the transmitted transverse displacement is the intensity barycenter of the transmission beam. Thus, it is a collective effect of the interaction between the light rays and the optical structure. The transverse shift will be determined by two factors. One is the optical structure itself, which determines the gradient of refractive index, and then determines the specific physical picture of spin orbit coupling when light is incident at a certain incident angle. The other is the incident beam. Its spot size, intensity distribution, especially the beam divergence, will affect the distribution of the transmitted field, and further affect the value of the transverse shift. For a Gaussian beam, through analytical method, we find that the value of the splitting is closely related to the ratio of the Fresnel transmission coefficient, while the optimal transmission coefficient ratio is related to the beam waist and incident angle. As for other types of beams, the formula of the transverse shift and its upper limit will be different. In theoretical analysis, the angular spectrum distribution needs to be adjusted according to the specific incident light. Besides, because the upper limit of the transverse displacement is a collective effect, even for a specific optical structure and at a certain incident angle, the transverse shift is still modulated by the incident beam, the upper limit may be obtained or not obtained. Therefore, at present, it is still hard to give a clear physical picture corresponding to the upper limit. In the following research, we should pay more attention to this issue.

Secondly, we anticipate that the reflected photonic SHE of layered structure should have the similar properties. The upper limit of the reflected SHE, as well as the corresponding conditions, can also be investigated using the same method. This expectation can be confirmed by the literature. For example, through mathematical analysis of Eq. (9) in the ref.^27^, it can be found that the reflected SHE also has an upper limit and the upper limit is the same as that of the transmission, ω_0_/2. In addition, when the Fresnel reflection coefficient $\frac{{\mathrm{r}}_{\mathrm{s}}}{{\mathrm{r}}_{\mathrm{p}}}={\pm k}_{0}{\omega }_{0}tan{\theta }_{i}-1$ is satisfied, the upper limit can be obtained. Thus, the upper limit properties of transmitted SHE and reflected SHE are perfectly unified.

Thirdly, the findings in this paper provide us a useful guide to enhance the photonic SHE. Exploring resonant optical structure or unconventional materials with a resonant Fresnel coefficient is helpful to obtain the upper limit. When light is incident upon an optical structure, if one of the P-polarized light and S-polarized light resonates, it will not only form a phase difference of π between the Fresnel coefficients, but also form a relatively large variation range of the ratio r. As a result, the upper limit of the transverse shift is easier to obtain. For example, it is known that both for reflection and transmission, the maximum shift is easier to be found near the Brewster angle. It is because that near this specific point, the transmission (reflection) coefficient of the p-polarized light experiences a resonance. Thus, the condition of $\mathrm{sin\varphi }=0$ is obtained, at the same time, a larger variation range of the Fresnel coefficient is also obtained. And further, if the condition of the optimal coefficient ratio is satisfied, the upper limit can be obtained. On the contrary, in many reported structures, the transverse shifts have already reached their upper limit^16,18^, further exploration of these structures would provide little improvement unless other incident beams are used.

Finally, the findings in this paper also provide us a useful guide to find enhanced photonic SHE with high efficiency. In field of photonic SHE, two factors are very important for practical applications. One is the transverse shift, and the other is the reflection or transmission efficiency. Usually, we need to make a trade-off between these two factors. However, according to our results, large transverse shift can be obtained when $\frac{{\mathrm{t}}_{\mathrm{p}}}{{\mathrm{t}}_{\mathrm{s}}}=\pm {\mathrm{k}}_{0}{\upomega }_{0}\mathrm{tan}{\uptheta }_{\mathrm{i}}-1$. We can explore an optical structure which excites a resonance at a proper incident angle ${\uptheta }_{\mathrm{i}}$. If the resonant incident angle ensures that the two transmission coefficients are comparable to each other, and both of them are relatively large, then large transverse shift and high efficiency can be obtained simultaneously. Besides, exploring optical structure possessing a near-unity transmission for one polarization while low transmission for the other polarization can also be a feasible method. For example, for the V-polarized incident light, if high transmittance is obtained for t_s_ and much low transmittance for t_p_, the condition of $\frac{{\mathrm{t}}_{\mathrm{p}}}{{\mathrm{t}}_{\mathrm{s}}}=\pm {\mathrm{k}}_{0}{\upomega }_{0}\mathrm{tan}{\uptheta }_{\mathrm{i}}-1$ can still be obtained at a relatively small incident angle. Therefore, larger transverse shift with near-unity efficiency can be obtained. The work reported in ref.^32^ belongs to this situation.

## Conclusion

In this paper, the transmitted photonic SHE through layered structures and materials is theoretically studied. At least two important conclusions are achieved. First, we find that there is an upper bound to the spin-dependent transverse shift, which is half of the incident beam waist. This finding offers a helpful guide for future research. Second, the relationship between the Fresnel transmission coefficients and the transverse shift is revealed. Unlike other investigations, we do not find a positive correlation between these two factors. In contrast, the optimal transmission ratio is determined by the incident angle and the beam waist. These findings will provide a deeper insight into the photonic spin Hall phenomena.

## Supplementary Information

Supplementary Information.
