# Supplementary material for: Limitations of the transmitted photonic spin Hall effect through layered structure
Source: Sci Rep. 2021 Oct 26;11:21083. doi: 10.1038/s41598-021-00681-0 (PMC8548548; doi:10.1038/s41598-021-00681-0)
Supplement: Supplementary file 1 — Supplementary Information. [file 41598_2021_681_MOESM1_ESM.pdf]

Supplementary information

Limitations of the transmitted photonic spin Hall effect through layered structure

Chong Miao[1], Dongxue Wang[1], Eric Herrmann[2], Zhiyuan Zheng[1], Haochong Huang[1], and Hua Gao[1, *]

[1] China University of Geosciences, School of Science, Beijing, 100083, China
[2] University of Delaware, College of Engineering, Department of Materials Science and Engineering, DE,19716, USA
[*] gaohua@cugb.edu.cn

Re derivation and verification of analytical expressions of the transverse shifts

Figure S1 illustrates a beam incident to a layered optical structure. The z axis of the laboratory Cartesian frame (x, y, z) is normal to the interfaces of the layered structure and the xz plane is the incident plane. We assume that the $+z_{i,\,t}$ direction of the local coordinate systems attached to the incident/transmitted beams is along the propagating direction of the central wave, as shown by the black lines. The red coordinates and the red lines are used for an arbitrary noncentral wave. We consider a monochromatic linearly polarized Gaussian beam passing through this layered structure. The angular spectrum of the incident Gaussian profile in the local coordinate system attached to the incident beam can be written as:

$$\tilde{E}_i(k_{ix}, k_{iy}) = \frac{\omega_0}{\sqrt{2\pi}} exp\,[-\frac{\omega_0^2\left(k_{ix}^2+k_{iy}^2\right)}{4}] \tag{S1}$$

where ω_0 is the beam waist.

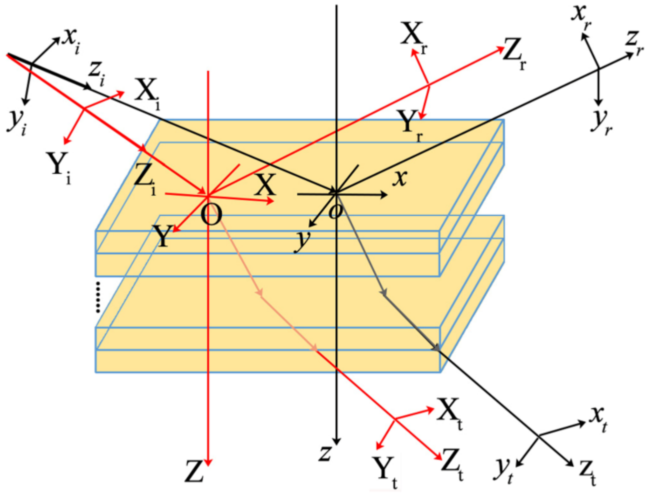

Figure.S1 Schematic illustrating the central and local wave vectors when a beam incident upon a layered structure.

In Eq.(S1), k_{ix} and k_{iy} are the transverse wave vectors of the noncentral waves. These transverse wave vector distributions will induce change of the incident angle for a noncentral wave. For example, if the incident angle of the central wave is θ_i, a small in-plane deflection of wave vector k_{ix} will induce an incident angle change to $\theta_i + \frac{\lambda}{2\pi} k_{ix}$. Considering the incident angle of each individual wave of the incident beam, and considering coordinate transformations between the local coordinate system attached to the incident beam and that attached to the transmitted beam, the relationship of the angular spectra between transmitted beam and incident beam in each local coordinate system can be obtained as[30]:

$$\begin{pmatrix} \tilde{E}_t^H \\ \tilde{E}_t^V \end{pmatrix} = \begin{pmatrix} t_p & \frac{k_{iy}}{k_0}\left(t_p - \frac{\cos\theta_t}{\cos\theta_i} t_s\right)\cot\theta_i \\ \frac{k_{iy}}{k_0}\left(\frac{\cos\theta_t}{\cos\theta_i} t_p - t_s\right)\cot\theta_i & t_s \end{pmatrix} \begin{pmatrix} \tilde{E}_i^H \\ \tilde{E}_i^V \end{pmatrix} \tag{S2}$$

where $\tilde{E}_t^H$, $\tilde{E}_t^V$, $\tilde{E}_i^H$ and $\tilde{E}_i^V$ are the H and V components of the angular spectra of the transmitted and incident beams, respectively. t_p and t_s denote the Fresnel transmission coefficients for H and V polarization states. In the following operation, these transmission coefficients, which will exist in the integrand functions, should be transformed into explicit functions of k_{ix} or k_{iy}. Using a Taylor series expansion and a first-order approximation, considering the symmetry of integrand function in the y direction, we only performed the Taylor expansion in the k_{ix} direction,

$$t_{p,s} = t_{p,s}(k_{ix} = 0) + \left.\frac{\partial t_{p,s}}{\partial k_{ix}}\right|_{k_{ix}=0} (k_{ix} - 0) \tag{S3}$$

where k_{ix}=0, k_{iy}=0 corresponds to the central wave of the incident beam. By substituting Eq.(S1) and Eq.(S3) into Eq.(S2), the angular spectrum of the transmitted field can be easily obtained. For an incident Gaussian beam with H polarization ($\tilde{E}_i^H = \tilde{E}_i$, $\tilde{E}_i^V = 0$), the transmitted angular spectrum components are:

$$\tilde{E}_t^H = (t_p + \frac{k_{ix}}{k_0}\frac{\partial t_p}{\partial_{\theta_i}})\tilde{E}_i \tag{S4}$$

$$\tilde{E}_t^V = [\frac{k_{iy}}{k_0}(\eta t_p - t_s)cot\theta_i] + \frac{k_{iy}k_{ix}}{k_0^2}(\eta\frac{\partial t_p}{\partial\theta_i} - \frac{\partial t_s}{\partial\theta_i})cot\theta_i]\tilde{E}_i \tag{S5}$$

where $\eta = \frac{\cos\theta_t}{\cos\theta_i}$. Usually, the whole structure is immersed inside one homogeneous medium. For simplicity, in the following deduction, η is set to 1. The complex amplitude of the transmitted beam in real space is then calculated via an inverse Fourier transform of the angular spectrum components. We obtain

$$E_t^H = (t_p + ix\frac{\frac{\partial t_p}{\partial\theta_i}}{(z_0+iz)}) \cdot E(x,y,z) \tag{S6}$$

$$E_t^V = \frac{i\,(\eta t_p - t_s)ycot\theta_i}{z_0+iz} \cdot E(x,y,z) - \frac{(\eta\frac{\partial t_p}{\partial\theta_i} - \frac{\partial t_s}{\partial\theta_i})cot\theta_i}{(z_0+iz)^2}xy \cdot E(x,y,x) \tag{S7}$$

where $\mathrm{E}(x,y,z) = \frac{z_0}{\omega_0(z_0+iz)}\exp(-\frac{k_0(x^2+y^2)}{2(z_0+iz)})$ is the complex amplitude distribution of the incident Gaussian beam, and $z_0 = k_0\omega_0^2/2$ is the Rayleigh length. Using a circular basis $\boldsymbol{e}_\pm = \frac{1}{\sqrt{2}}(\boldsymbol{e}_x \pm i\boldsymbol{e}_y)$, the transmitted complex amplitude components can be decomposed into two circular components as:

$$E_H^{\pm} = \frac{1}{2}[t_p + ix\frac{\frac{\partial t_p}{\partial\theta_i}}{z_0+iz} \mp y\frac{(\eta t_p - t_s)cot\theta_i}{z_0+iz} \mp \frac{ixy(\eta\frac{\partial t_p}{\partial\theta_i} - \frac{\partial t_s}{\partial\theta_i})cot\theta_i}{(z_0+iz)^2}] \cdot E(x,y,z) \tag{S8}$$

At any given plane, z=const, the transverse displacements of each circular component of the transmitted beam are defined as:

$$\delta_{H,V}^{\pm} = \frac{\iint y|E_H^{\pm}|^2 dxdy}{\iint|E_H^{\pm}|^2 dxdy} \tag{S9}$$

In our calculations, the parity of the integral factor and the Poisson integral formula are used in this step. Through careful calculation, the analytical expressions of the transverse displacements can be obtained as:

$$\delta_H^{\pm} = \pm\frac{k_0\omega_0^2 cot\theta_i[|t_p|^2 - Re(t_p \cdot t_s{}^*) + z/z_0\,Im(t_p \cdot t_s{}^*)]}{k_0{}^2\omega_0^2|t_p|^2 + |t_p - t_s|^2 cot^2\theta_i + \frac{\partial t_p}{\partial\theta_i}\cdot\frac{\partial t_p}{\partial\theta_i}^*} \tag{S10}$$

Similarly, the transverse displacements with V polarization input can be obtained by making replacements of $t_p \rightarrow t_s$, $t_s \rightarrow t_p$ in Eq.(S10),

$$\delta_V^{\pm} = \pm\frac{k_0\omega_0^2 \cot\theta_i[|t_s|^2 - Re(t_s \cdot t_p{}^*) + z/z_0\,Im(t_s \cdot t_P{}^*)]}{k_0^2\omega_0^2|t_s|^2 + |t_s - t_p|^2 cot^2\theta_i + \frac{\partial t_s}{\partial\theta_i}\cdot\frac{\partial t_s^*}{\partial\theta_i}} \tag{S11}$$

The displacements contain both transverse spatial shifts and an angular spin shift, where the z dependent term is the angular spin shift. For actual SHE structures, the thicknesses of the optical structures are usually much smaller than the Rayleigh length z_0 of the light source,

thus, the angular spin shift can be ignored. Here we only consider the spatial shifts. Eq.(S10) and Eq.(S11) are similar to many expressions of the transverse displacements in the literature, but they are not exactly the same. Formally, these two formulae are closest to the expressions in Ref. [10] and Ref. [22], except that the last term of the denominator is $\frac{\partial t_{s,p}}{\partial \theta_i} \cdot \frac{\partial t_{s,p}^*}{\partial \theta_i}$ rather than $\left(\frac{\partial t_{s,p}}{\partial \theta_i}\right)^2$. It is apparent that when $t_{s,p}$ is a complex number, these two terms are different, especially when $t_{s,p}$ varies rapidly with θ_i.

To distinguish which formula is correct, we used them separately to study an ENZ-air-ENZ structure. The permittivity of the three layers are set to be 0.01, 1, 0.01 in turn and the corresponding thicknesses take values of λ, 3λ, λ. The wavelength and the beam waist of the incident beam are set to be λ=0.633 μm and ω_0=10λ, respectively. Fig.S2 gives the calculated transverse shifts, where Fig.S2 (a) presents the results calculated by using $\frac{\partial t_{s,p}}{\partial \theta_i} \cdot \frac{\partial t_{s,p}^*}{\partial \theta_i}$ while Fig.S2 (a) corresponds to $\left(\frac{\partial t_{s,p}}{\partial \theta_i}\right)^2$. Apparently, at some large resonant angles, the transverse shifts in Fig.S2 (b) are much greater than those in Fig.S2 (a). Moreover, if we increase the beam waist to 50λ, the largest transverse shift in Fig.S2 (b) almost reaches one thousand times of λ. It is apparently unreasonable.

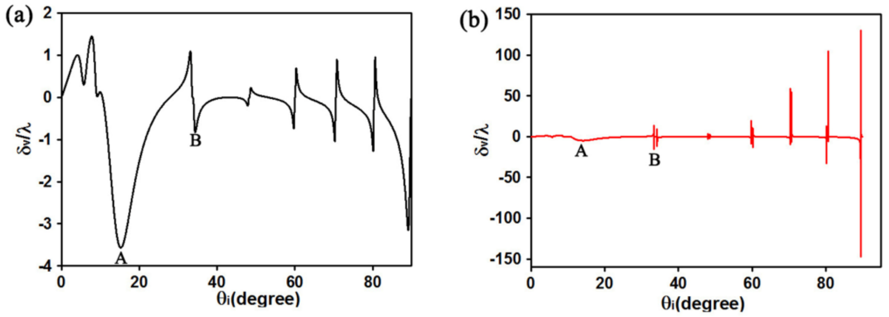

Fig.S2 The transverse shifts calculated using $\frac{\partial t_{s,p}}{\partial \theta_i} \cdot \frac{\partial t_{s,p}^*}{\partial \theta_i}$ (a) and using $\left(\frac{\partial t_{s,p}}{\partial \theta_i}\right)^2$ (b) for V-polarized light, where all the parameters are identical to each other.

We also used a commercial software COMSOL Multiphysic to simulate the interaction between a Gaussian beam and this three-layer structure. In simulation all the parameters of the incident beam and the optical structure are exactly the same as those used in Fig.S2. Fig.S3 shows the electric field distributions in the plane 0.1 μm from the exit surface for three different incident angles, θ_i=0, 15.3, 34 degree. It is seen that, for normal incidence, θ_i=0 degree, the field distribution almost remains unchanged compared with the incident Gaussian

beam, indicating that there is no photonic SHE for normal incidence. We swept the parameter of the incident angle, and the sweep step is modulated to be small enough to avoid missing the resonant peak. It is found that the largest transverse shift appears at θ_i=15.3 degree, shown as Fig.S3(b), corresponding to the point A in Fig.S2. Fig.S3 (c) is the electric distribution at θ_i=34 degree, corresponding to the point B in Fig.S2. The light spot is elongated along the y direction, however, the separation is much smaller than that at θ_i=15.3 (point A), this is obviously contrary to the calculated results in Fig.S2(b). Therefore, only the results calculated by using $\frac{\partial t_{s,p}}{\partial \theta_i} \cdot \frac{\partial t_{s,p}^*}{\partial \theta_i}$ are self-consistent. The transverse displacements calculated by using Eq.(S10) and Eq.(S11) are reliable.

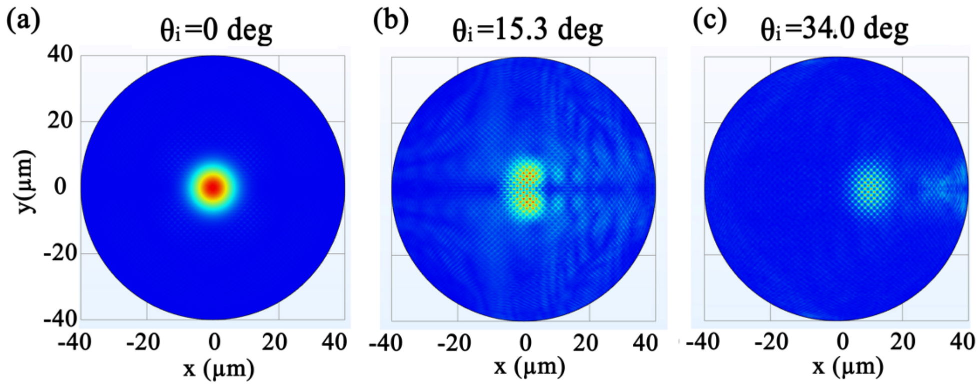

Fig.S3 The electric field distribution 0.1 μm from the exit surface for different incident angles: (a) θ_i=0 degree, (b) θ_i=15.3 degree, (c) θ_i=34.0 degree
